# Phylogenetic Relatedness and Genome Structure of *Yersinia ruckeri* Revealed by Whole Genome Sequencing and a Comparative Analysis

**DOI:** 10.3389/fmicb.2021.782415

**Published:** 2021-11-18

**Authors:** Mostafa Y. Abdel-Glil, Uwe Fischer, Dieter Steinhagen, Una McCarthy, Heinrich Neubauer, Lisa D. Sprague

**Affiliations:** ^1^Friedrich-Loeffler-Institut, Institute of Bacterial Infections and Zoonoses (IBIZ), Jena, Germany; ^2^Friedrich-Loeffler-Institut, Institute of Infectiology, Greifswald-Insel Riems, Germany; ^3^Fish Disease Research Unit, Institute for Parasitology, University of Veterinary Medicine Hannover, Hanover, Germany; ^4^Marine Laboratory, Marine Scotland, Aberdeen, United Kingdom

**Keywords:** *Yersinia ruckeri*, genome, taxonomy, evolution, fish, enteric redmouth disease, phylogeny, cgMLST and SNPs

## Abstract

*Yersinia ruckeri* is the causative agent of enteric redmouth disease (ERM), a serious infection that affects global aquaculture with high economic impact. The present study used whole genome sequences to perform a comparative analysis on 10 *Y. ruckeri* strains and to explore their genetic relatedness to other members of the genus. *Y. ruckeri*, *Yersinia entomophaga*, and *Yersinia nurmii* formed a species complex that constitutes the most basal lineage of the genus. The results showed that the taxonomy of *Y. ruckeri* strains is better defined by using a core genome alignment and phylogenetic analysis. The distribution of accessory genes in all *Yersinia* species revealed the presence of 303 distinctive genes in *Y. ruckeri*. Of these, 169 genes were distributed in 17 genomic islands potentially involved in the pathogenesis of ERM via (1) encoding virulence factors such as Afp18, Yrp1, phage proteins and (2) improving the metabolic capabilities by enhancing utilization and metabolism of iron, amino acids (specifically, arginine and histidine), and carbohydrates. The genome of *Y. ruckeri* is highly conserved regarding gene structure, gene layout and functional categorization of genes. It contains various components of mobile genetic elements but lacks the CRISPR-*Cas* system and possesses a stable set of virulence genes possibly playing a critical role in pathogenicity. Distinct virulence plasmids were exclusively restricted to a specific clonal group of *Y. ruckeri* (CG4), possibly indicating a selective advantage. Phylogenetic analysis of *Y. ruckeri* genomes revealed the co-presence of multiple genetically distant lineages of *Y. ruckeri* strains circulating in Germany. Our results also suggest a possible dissemination of a specific group of strains in the United States, Peru, Germany, and Denmark. In conclusion, this study provides new insights into the taxonomy and evolution of *Y. ruckeri* and contributes to a better understanding of the pathogenicity of ERM in aquaculture. The genomic analysis presented here offers a framework for the development of more efficient control strategies for this pathogen.

## Introduction

The genus *Yersinia* currently comprises 26 species with different ecological habitats and pathogenicity. *Yersinia ruckeri* is a Gram negative, rod shaped, facultative intracellular enterobacterium and the causative agent of enteric redmouth disease (ERM), a serious septicaemic bacterial disease of salmonids. Fish in all stages of development are susceptible, resulting in a high degree of mortality (Guijarro et al., 2018). The clinical picture is characterized by hemorrhages in the skin and mucosa, as well as exophthalmia, darkening of the skin, inflammation of the lower intestine and splenomegaly (Kumar et al., 2015). Phenotypically, strains can be distinguished according to their biotypes (biotype 1 and biotype 2), serotypes (O1a, b; O2 a, b, c; O3; and O4) as well as their outer membrane protein (OMP) profiles (Romalde and Toranzo, 1993; Kumar et al., 2015; Wrobel et al., 2020). OMPs contribute significantly to the composition of the outer membrane of Gram-negative bacteria and play a major role in virulence. Studies on the composition of the outer membrane proteome of *Y. ruckeri* isolates from rainbow trout and Atlantic salmon revealed a high degree of variation between strains (Ormsby et al., 2019; Ormsby and Davies, 2021). Several pathogenicity factors have been implicated in the virulence of *Y. ruckeri* strains, including toxins, secretion systems, iron and amino acid utilization systems, lipopolysaccharides, flagella and plasmids. The current knowledge on virulence factors and infection pathways of *Y. ruckeri* is outlined in recently published reviews (Guijarro et al., 2018; Wrobel et al., 2019).

*Yersinia ruckeri* poses a serious threat to the global aquaculture industry and infection can result in significant economic losses. Disease management is currently based on vaccination and antibiotic treatment. The first commercial fish vaccine for ERM was a bacterin prepared from formalin inactivated whole cells of *Y. ruckeri* and licensed in 1976. However, outbreaks are being increasingly reported throughout the world (Kumar et al., 2015; Wrobel et al., 2019). This development of “vaccine breakthrough” strains necessitates the design of new and more effective vaccines based on data obtained from comparative genome studies. Moreover, these studies can elucidate how fish pathogens spread and evolve with aquaculture, help to create management strategies to improve biosecurity and ultimately significantly reduce financial losses (Ormsby and Davies, 2021; Yang et al., 2021).

Genome comparison studies enable detailed insights into understanding pathogenicity and evolution of bacteria and help to clarify transmission routes as well as the geographic spread of pathogens. In this study, (1) we describe the genomic features of ten circularized genomes of *Y. ruckeri* strains of mostly clinical origin; (2) explore the genetic relatedness of *Y. ruckeri* within the genus *Yersinia* by comparing our data with the genomic data of 90 representative strains of all validly published *Yersinia* species; (3) describe the comparative phylogenetic analysis and *in silico* virulence profiling of the ten sequenced strains with published genomes of 67 *Y. ruckeri* strains.

## Results

### *Yersinia ruckeri* Strains and Genome Sequencing

Ten *Y. ruckeri* strains of predominantly clinical origin, i.e., diseased fish were characterized by traditional bacteriological methods and sequenced using Pacific Bioscience and Illumina MiSeq platforms. These included seven strains from Germany isolated between 2005 and 2011, two strains isolated in Scotland in 1999 and 2007, and the *Y. ruckeri* type strain (DSM18506/ATCC 29473; here: 16Y0180) (Tables 1, 2).

**TABLE 1:** List of *Yersinia ruckeri* strains sequenced in this study.

Strain number	Alias	Host	Isolation Year	Origin
16Y0180	Type strain (DSM18506/ATCC 29473)	Rainbow trout	–	Idaho, United States
17Y0153	G1S1	Rainbow trout	2008	North Rhine-Westphalia, Germany
17Y0155	LT13-1/0811	Rainbow trout	2011	North Rhine-Westphalia, Germany
17Y0157	178-1/05	Brown trout	2005	Lower Saxony, Germany
17Y0159	285-1/05	Pike	2005	Lower Saxony, Germany
17Y0161	111-1/05	–	2005	Lower Saxony, Germany
17Y0163	1521/11	Rainbow trout	2011	Hesse, Germany
17Y0189	KP6	Rainbow trout	2011	North Rhine-Westphalia, Germany
17Y0412	MT2209	Atlantic salmon	1999	Highlands, Scotland, United Kingdom
17Y0414	MT3187	Atlantic salmon	2007	Western Isles, Scotland, United Kingdom

**TABLE 2:** Phenotypic characterization of the ten *Yersinia ruckeri* strains.

Strain number	Biochemical identification	CO	Catalase	Gram staining	Morphology	Motility	API 20E	Glucose/Gas	Tween 80	Tween 20	Xylose	Nitrate	Gelatinase	Citrate	VP	MR	Sorbitol	OF-O/F	N2
16Y0180	*Y. ruckeri*	−	+	−	rods	+	1105100	+	nd	nd	−	nd	−	−	+	nd	−	nd	nd
17Y0153	*Y. ruckeri*	−	+	−	short rods	−	530710017	+/−	−	−	−	+	+	+	+	+	−	+/+	nd
17Y0155	*Y. ruckeri*	−	+	−	short rods	+	530710057	+/−	+	+	−	+	+	−	+	+	−	+/+	nd
17Y0157	*Y. ruckeri*	−	+	−	short rods	+	530750057	+/−	+	+	−	−	+	+	+	−	+	+/+	+
17Y0159	*Y. ruckeri*	−	+	−	rods	+	530570057	+/−	+	+	−	+	−	+	+	−	+	nd	nd
17Y0161	*Y. ruckeri*	−	+	−	short rods	+	530750047	+/−	+	+	−	+	+	+	+	−	+	+/+	nd
17Y0163	*Y. ruckeri*	−	+	−	short rods	+	530750057	+/−	+	+	−	+	+	+	+	−	+	+/+	nd
17Y0189	*Y. ruckeri*	−	+	−	rods	−	5107100	+	nd	nd	−	nd	+	−	+	nd	−	nd	nd
17Y0412	*Y. ruckeri*	−	+	−	rods	+	5107100	+	nd	nd	−	nd	+	−	+	nd	−	nd	nd
17Y0414	*Y. ruckeri*	−	+	−	rods	+	5107100	+	nd	nd	−	nd	+	−	+	nd	−	nd	nd

*CO: cytochrome oxidase reaction; VP: Voges-Proskauer test; MR: methyl red test; OF-F/O: Oxidation-Fermentation; nd: not determined.*

Genome sequencing using the Pacific Bioscience SMRT^®^ method produced on average 80,586 reads (70592–92078) and 900,928,849 bases (632,133,554–1.276,956,896) per strain with a mean sequencing depth of 178-fold (129 to 248-fold). The mean read length was 9,065.3 bp (1007–14225 bp) and the mean N50 value of the reads was 14929.9 bp (10855–19303 bp) (Supplementary Table 1). Sequencing using Illumina MiSeq generated on average 1,371,836 paired-end reads (664,572–2.680,262) and 364,034,507 bases (180,017,799–686,936,084) accounting for a mean theoretical sequencing depth of 98.4-fold (48.6 to 185.6-fold) based on the length of the reference *Y. ruckeri* genome, Big Creek 74 (accession GCF000964565.1) (Supplementary Table 1).

### Overview of Features of Circularized *Y. ruckeri* Genomes

#### Genome Description

Sequenced genomes were assembled *de novo* and the resulting circularized genomes were iteratively polished with Illumina reads to obtain finished genomes with high fidelity (see section “Materials and Methods”). The complete genome of *Y. ruckeri* revealed a single circular chromosome with several distinct features (Supplementary Figure 1). The structure of the chromosomes was characterized by a high level of conservation between the strains with regard to genome length, GC content, RNA genes and coding capacity. The size of the chromosome ranged between 3.67 and 3.81 mega bases (Mb) with an average GC content of 47.5% (Supplementary Table 1). The density of coding DNA sequences (CDS) averaged 84.2% (84.02–84.46%) with total annotated CDS features averaging 3374 (3267–3460) per genome and average gene size of 939 bases (90–12,159 bp). The chromosome of *Y. ruckeri* contained 81–84 tRNA genes and seven ribosomal RNA operons comprising 16S, 23S, and 5S rRNA genes (Supplementary Table 1). The genome of the *Y. ruckeri* strains exhibited a comparatively high number of pseudogene candidates, which comprised on average 5.7% (4.4–7.1%) of the total number of coding sequences (Supplementary Table 1). Of the predicted pseudogenes, 40–50% of the pseudogenes represented fragmented CDS while 17–24% represented truncated genes.

#### Gene Layout

**FIGURE 1:**
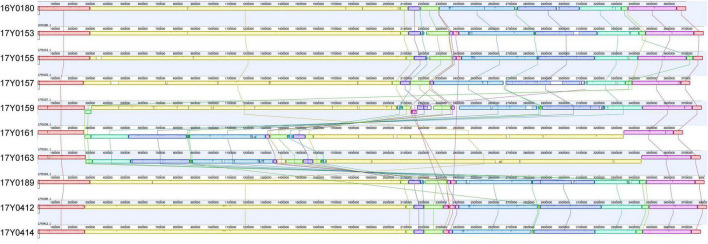
Multiple genome alignment with progressiveMauve for the 10 *Yersinia ruckeri* strains relative to the type strain (16Y0180; DSM 18506); Syntenic homologous regions between strains are shown as colored blocks with a similarity profile corresponding to the average conservation of sequences within the blocks. Homologous blocks are displayed in matching colors and connected by vertical lines. Blocks placed below the center line are in inverse orientation. Regions outside the blocks represent unique DNA regions in the genomes. The scale is in base pairs.

A comparison of the gene layout in the chromosome of the ten strains revealed a collinear genome with a limited frequency of gene rearrangements in the majority of strains. In two strains, 17Y0161 and 17Y0163, a large segment of the chromosome (∼3 Mb) was symmetrically inverted relative to the replication fork (Figure 1) and was flanked by rRNA operons.

#### Functional Annotation of Genes

Functional annotation of the chromosomal genes using the Clusters of Orthologous Groups (COGs) classification scheme showed no significant variation between the distribution patterns of the COG groups among the genomes (standard deviation per COG group between genomes ranged from 0.8 to 12 genes; Supplementary Table 2). More than 90% of the genes were assigned to a specific COG group including the 18% of genes belonging to the COG category “S; function unknown.” Of the genes annotated with known functions, the majority (45–47%) belonged to the “metabolism” group followed by the “cellular processes and signaling” (29–30%) and the “information storage and processing” (23–24%) groups (Supplementary Table 2). Some COG groups occurred with higher frequency such as the “amino acid transport and metabolism; group E” (20%), the “transcription, cell wall/membrane/envelope biogenesis; group M” (17%) and the “intracellular trafficking, secretion, and vesicular transport; group U” (15–17%). Contrariwise, only a few genes (∼2%) were assigned to the COG group “defense mechanisms” (Supplementary Table 2).

#### Clustered Regularly Interspaced Short Palindromic Repeats (CRISPR) and Mobile Genetic Elements

No CRISPR-*Cas* regions were detected in any of the sequenced genomes. Therefore, the mobile element structures in the genomes i.e., plasmids, phages and insertion sequences were analyzed in depth. Seven of the ten genomes contained plasmids; six genomes contained a single plasmid and one genome three plasmids (Supplementary Table 1). By applying the Hadamard matrix, which combines values of percentage identity and alignment length between plasmid genome pairs, the nine plasmids could be allocated to six plasmid types. A 103 kb plasmid was present in four genomes while the other three genomes carried distinct plasmids of 23.8, 28.7, 59.4, 83.7, and 85.1 kb, respectively. The number of coding sequences varied between 25 and 101 CDS per plasmid. Most of the plasmid genes encoded hypothetical proteins. The COG annotation revealed that the function of 30% of the well-characterized plasmid genes was related to “replication, recombination and repair.” Functions related to metabolism were poorly represented in the plasmid genes (Supplementary Table 3).

Integrated and complete phages were predicted in the chromosome of nine strains based on PHASTER classification with a phage completeness score between 100 and 150 (Arndt et al., 2016); six strains were predicted to contain two phages, two strains to contain three phages and one strain to contain four phages (Table 3). Nine phage types were predicted in total. Each analyzed *Y. ruckeri* strain displayed a unique combination of prophages (Table 3). However, the phage “Salmon 118970 sal3” (NC_031940) from the *Myoviridae* family was present in eight strains (Table 3). Salmon 118970 sal3 has been reported in different bacterial species such as *Salmonella* Typhimurium and *Escherichia coli*, and was found to exhibit lytic activity against *Salmonella* strains (Paradiso et al., 2016). The GC content of the predicted phages was generally different to that of the *Y. ruckeri* genome, ranging between 45.1 and 49.8%.

**TABLE 3:** Summary of mobile genetic elements in the complete genomes of the ten *Yersinia ruckeri* strains.

	Insertion sequences*										Intact phages in the chromosome (number, GC content%)**									
Strain number	IS1	IS200/IS605	IS256	IS3	IS481	IS5	ISL3	new	Total number	Percentage	GF_2 NC_026611	SfI NC_027339	SfV NC_003444	ENT90 NC_019932	500465_1 NC_049342	SuMu NC_019455	118970_sal3 NC_031940	SEN34 NC_028699	PY54 NC_005069	Length of predicted phages
16Y0180		3	1	21					25	0.94			1 (49.8%)		1 (46.2%)					33.4- and 35.4-kb
17Y0153		3	1	18				1	23	0.84			1 (49.8%)				1 (47.1%)			33.3- and 66.5-kb
17Y0155		3		18					21	0.79		1 (49.8%)					1 (47.8%)			33.4- and 51-kb
17Y0157	1	3	1	11					16	0.56										
17Y0159		1	5	12	1		5		24	0.85					1 (45.1%)	1 (49.2%)	1 (47.2%)			53. 5-, 43. 4-, and 38.9-kb
17Y0161		2		9		1			12	0.46				1 (49.2%)			1 (47.8%)			47.9- and 46.4-kb
17Y0163		3		6					9	0.35							1 (48.8%)	1 (48.7%)		45.9- and 30.8-kb
17Y0189		3		18					21	0.79							2 (47.1 and 49.8%)			51- and 33.3-kb
17Y0412		3	1	14	1				19	0.7	1 (47.3%)					1 (48.9%)	1 (48.5%)		1 (48.5%)	35. 6-, 41. 9-, 44. 9-, and 50.1-kb
17Y0414		3	1	14					18	0.64	1 (47.4%)						1 (48.5%)		1 (48.5%)	34. 1-, 44. 9-, and 50.1-kb

*Only complete insertion sequences are reported. **PHASTER completeness score 100–150.

Nine to twenty-five insertion sequences (IS) between 13 and 34 kb in size were predicted for each chromosome (Table 3), representing a variety of IS families, i.e., IS1, IS200/IS605, IS256, IS3, IS481, IS5, and ISL3. Most of the observed IS elements belonged to the IS3 family accounting for 50 to 85% of the total number of insertion sequences per genome (Table 3).

### Comparative Analysis of *Y. ruckeri* to the Genus *Yersinia*

In order to explore the genetic relationship of the *Y. ruckeri* strains with the genus *Yersinia*, we additionally downloaded the genome sequence data of 90 strains representing the 26 currently validated *Yersinia* species (Savin et al., 2019; Le Guern et al., 2020; Nguyen et al., 2021). Reference genomes in the RefSeq database and the genomes of the species type strains were prioritized during data retrieval from NCBI. The genomic features of the downloaded WGS data are summarized in Supplementary Table 4.

#### 16S rRNA Gene Analysis

The rRNA genes were extracted from the ten sequenced *Y. ruckeri* genomes. The rRNA genes were present in seven almost identical copies across the genome with a sequence similarity of 99–100%. Phylogenetic analyses of the 16S rRNA region of the 10 *Y. ruckeri* strains and the representative *Yersinia* species (*n* = 88) showed a clear separation of *Y. ruckeri* from the other *Yersinia* species of the genus, with all *Y. ruckeri* strains clustering in a monophyletic lineage (Supplementary Figure 2). The 16S rRNA genes of *Y. ruckeri* were highly conserved and showed more than 99.1% sequence identity (Supplementary Table 5). Based on 16S rRNA gene analysis, the closest related species to *Y. ruckeri* is *Yersinia kristensenii* with an average nucleotide similarity of 99% (Supplementary Table 5).

#### Genomic Average Nucleotide Identity

**FIGURE 2:**
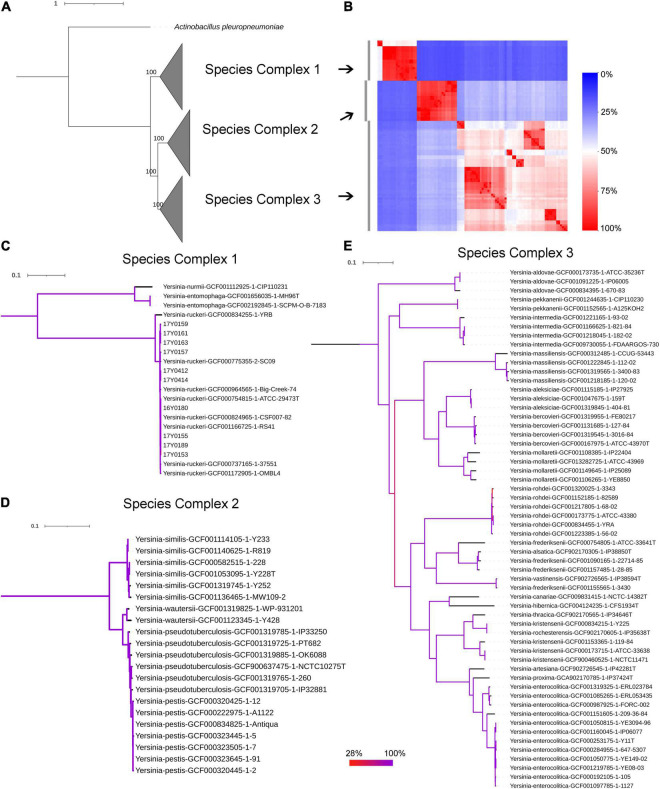
Phylogenetic relationships within the genus *Yersinia*. **(A)** Maximum likelihood (ML) phylogenetic analysis based on the core protein alignment with the inclusion of strain *Actinobacillus pleuropneumoniae* as an outgroup strain (strain S4074, accession NZ_CP030753.1). **(B)** A heat map showing the alignment coverage for nucleotide identity calculation. **(C–E)** ML phylogenetic analysis based on the core nucleotide alignment of 2,002 genes (2,046,925 bp) present in more than 95% of the investigated 100 strains. The panels **(C–E)** correspond to species complex 1, 2, and 3, respectively. Branch coloration refers to the bootstrap support based on the analysis of 100 resampled trees.

The genomic average nucleotide identity (gANI) was estimated by performing a pairwise alignment of genome stretches with MUMmer (Pritchard et al., 2016). The ANI values between the *Y. ruckeri* strains averaged 99% which exceeded the 95–96% ANI cut-off generally used to define species boundaries (Richter and Rossello-Mora, 2009; Supplementary Figure 3A). In contrast, ANI values between the *Y. ruckeri* strains and the different *Yersinia* species averaged around 84% nucleotide similarity (Supplementary Figure 3B). The alignment fraction used to estimate nucleotide identity, ranged between 15 and 100% (Supplementary Figure 3C). Sequence alignment fractions encompassing more than 50% of the genome grouped the 26 *Yersinia* species into three distinct species complexes (Supplementary Figure 3C and Figure 2). *Y. ruckeri*, *Yersinia entomophaga*, and *Yersinia nurmii* form species complex 1 while species complex 2 contains *Yersinia similis*, *Yersinia pseudotuberculosis*, *Yersinia pestis*, and *Yersinia wautersii*. Species complex 3 includes all other *Yersinia* species (*n* = 19) (Figure 2). The independent ANI algorithms FastANI and Microbial Species Identifier (MiSI) corroborated these results (Supplementary Table 6).

#### Core-Genome-Based Phylogeny

The soft-core genome encompasses 2,002 genes (2,046,925 bp) present in over 95% of the *Yersinia* genomes. The consensus SNP positions comprise 25% (*n* = 512,502) of the concatenated gene alignments, with 96% (*n* = 491,746) of them parsimony-informative. The phylogenetic analysis based on the core nucleotide alignment was performed by inferring a mid-point rooted maximum-likelihood tree (Figure 2). The branching pattern of the phylogeny was consistent with the ANI results, showing that the species of this genus grouped into three monophyletic lineages corresponding to the three species-complexes (Figure 2), with *Y. ruckeri* belonging to species complex 1. This species complex constitutes the most basal clade that roots the *Yersinia* genus (Figure 2).

#### Core Genome Multilocus Sequence Typing Allele Typing

**FIGURE 3:**
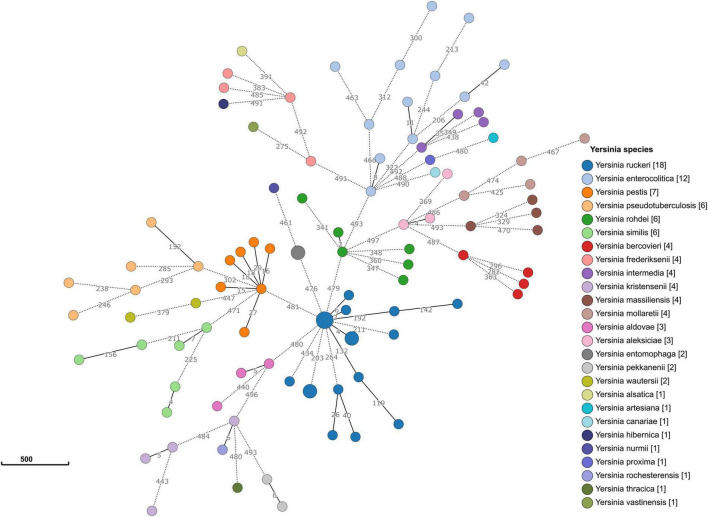
Minimum spanning tree of the genus-based cgMLST for *Yersinia* strains. The dataset includes 90 representative genomes of 26 different *Yersinia* species as well as the ten strains sequenced in the study. Allelic profiles were calculated using the Genome Comparator function of BIGSdb. Nodes (circles) were colored according to species. Each node corresponds to a single core genome ST. The size of the node corresponds to the number of strains. The number of different alleles is shown on the branches (lines) between the nodes. Branches represented by dotted lines indicate more than 200 allelic differences between the strains.

Allele typing of the core genome was applied to our data (February 2021) using the recently published *Yersinia* core genome multilocus sequence typing (cgMLST) scheme (Savin et al., 2019). Two genomes (17Y0153 and 17Y0155) were assigned to the core-genome sequence type (cgST) 435. The results also showed that 13 of the 500 cgMLST loci were consistently absent in all ten *Y. ruckeri* genomes. A minimum spanning tree based on the allelic profiles of the 100 analyzed *Yersinia* strains revealed long allelic distances for the 500 loci among the genomes (Figure 3). Distance thresholds defining a *Yersinia* species on genus-based cgMLST were found to vary greatly between the species as previously described (Savin et al., 2019). Based on allele typing of the cgMLST scheme, the *Y. ruckeri* genomes grouped together, with pairwise allelic differences varying between 2 and 434. However, the allelic distances to the neighboring species were 476 to *Y. entomophaga*, 479 to *Yersinia rohdei*, 480 to *Yersinia aldovae* and 481 to *Y. pestis*.

#### Distribution of Accessory Genes

Next, we sought to identify the genes that are significantly associated with the *Y. ruckeri* species. We calculated a pangenome of 25,498 genes for the 100 *Yersinia* strains, of these 23,496 corresponded to accessory genes. A pan-genome-wide association study (GWAS) approach identified 34 genes significantly underrepresented in *Y. ruckeri* compared to the other *Yersinia* species (100% sensitivity and specificity, see section “Materials and Methods”) (Supplementary Table 7). The missing genes coded predominantly for putative transporter proteins. Contrariwise, 303 genes had a statistically significant association with *Y. ruckeri* and their uniqueness was confirmed using BLASTN at 90% identity and 80% coverage (Supplementary Table 7). The COG annotation assigned the majority of genes to metabolism (*n* = 103), cell wall/membrane/envelope biogenesis (*n* = 30), intracellular trafficking, secretion and vesicular transport (*n* = 26), transcription (*n* = 20), and cell motility (*n* = 11) (Supplementary Table 8). Ninety-three genes were predicted to encode cytoplasmic proteins, 113 to encode cytoplasmic membrane proteins, 4 to encode extracellular proteins.

Of the 303 *Y. ruckeri*-specific genes, 169 were arranged into 17 gene clusters/regions (1.5–49.6 kb). The largest genomic region (49 kb) included genes predominantly involved in protein utilization e.g., the *Y. ruckeri* protease 1 (Yrp1)-encoding operon that comprises *yrp1*, encoding for serralysin metalloprotease (EC 3.4.24.40), *inh* for a protease inhibitor and *yrpD*, *yrpE*, and *yrpF* for type I ABC protease exporters. In addition, genes encoding for histidine utilization proteins were predicted including EC 3.5.1.68 (N-formylglutamate deformylase), EC 3.5.2.7 (Imidazolonepropionase), histidine utilization repressor, EC 3.5.3.13 (Formiminoglutamic iminohydrolase), and HutD. Furthermore, an operon encompassing five successive genes putatively involved in the arginine succinyltransferase (AST) pathway for arginine metabolism, were predicted including EC 2.6.1.81 (succinylornithine transaminase), EC 2.3.1.109 (arginine N-succinyltransferase), EC 1.2.1.71 (succinylglutamate-semialdehyde dehydrogenase), EC 3.5.3.23 (N-succinylarginine dihydrolase), and EC 3.5.1.96 (succinylglutamate desuccinylase). A gene encoding an OMP complex (OMPC) was found between the arginine and histidine utilization operons. No mobile elements were detected in this large genomic region.

The virulence gene of the antifeeding prophage 18 (*Afp18*) was detected in the second largest genomic region (30 kb). Further downstream, the transposase gene *IS285* bordered *Afp18*. Three additional genes with similar “left” orientation bordered by a transposase gene, ISKpn20 of the IS3 family, were found upstream of *Afp18* suggesting a putative operon structure. In the type strain, the *Y. ruckeri* invasin-like molecule (yrIlm) was located upstream of this genomic region and flanked by the ISEc39 transposase gene of the IS256 family. Phage proteins representing an incomplete phage of 7.7 kb were also predicted. This incomplete phage was similar to the Bacill_BCD7 phage (NC_019515).

The third genomic island (30 kb) included several genes encoding for a phosphotransferase system (PTS) involved in the uptake of carbohydrates (transport of N-acetylgalactosamine) by catalyzing the phosphorylation of sugar substrates. Three genes encoding sialidases flanked this region; two genes for N-acetylneuraminate epimerase (EC 5.1.3.24) and one gene for N-acetylneuraminic acid outer membrane channel protein NanC. An ISSpr1 transposase of the IS3 family was located downstream of the sialidase genes. A PTS system was additionally predicted in a further 25 kb genomic island. The latter harbored a cold-shock protein belonging to the CSP family.

A 21 kb gene cluster was detected comprising 15 genes, primarily involved in iron transport including a catechol siderophore system known as ruckerbactin, an important pathogenicity factor for *Y. ruckeri* infection in fish. A further 20 kb-genomic region was flanked by TnBth2 transposase of the Tn3 family. This region included three homolog genes to the SsrAB two-component regulatory system present within the *Salmonella* pathogenicity island (SPI-2). SsrAB is essential for survival and replication within host immune cells. Two type II secretion systems previously described in the genome of *Y. ruckeri* strain SC09 were detected in association with two genomic regions; one thereof was flanked by an ISSpr1 transposase gene. However, the two T2SS systems were not exclusively present in *Y. ruckeri* genomes. Supplementary Table 9 lists the composition and predicted annotation function of the identified *Y. ruckeri*-specific gene clusters.

### Comparative Analysis of *Y. ruckeri*

#### *Y. ruckeri* Pangenome

To explore the diversity of the species *Y. ruckeri*, the sequence data of the ten *Y. ruckeri* strains sequenced in this study were combined with 67 *Y. ruckeri* genomes from prior studies (Supplementary Table 10). A total of 263,461 protein-coding sequences was present in the 77 strains. The pangenome analysis clustered the coding sequences into 5,655 gene clusters corresponding to the entirety of non-redundant genes. The core genome comprised 52% of the pangenome genes; 2,955 genes (2,892,182 bp) were present in more than 95% of the strains and 2,752 genes were present in all strains. A divergence of 2.4% was observed in the core genome for *Y. ruckeri*. SNP sites in the core orthologous genes covered 68.5 kb. The accessory genome comprised 2,700 genes distributed in less than 95% of the strains. Of these, 794 genes were strain-specific i.e., exclusively present in one strain. The pangenome was in its open form as previously demonstrated (Barnes et al., 2016). The trajectory pattern of the curve depicting the pangenome increased by adding more genomes. This openness was confirmed (Bpan = 0.25) by applying the regression models proposed by Tettelin et al. (2008). A limited reduction in the size of the core genome was observed. The core genome accounted for 85.3% of the coding sequences in the *Y. ruckeri* genome (n. CDS = 3,225, Big Creek 74, NZ_CP011078.1), while the core versus pangenome ratio was 48.6%. Supplementary Figure 4 shows the trending pattern of the pangenome increase versus core genome decrease with sequential addition of genomes. The curve representing the new gene detection did not converge to zero, with new genes continuously contributing to the expansion of the pangenome.

#### Core Genome Phylogeny

Parsnp alignment followed by Gubbins-based filtration of loci with elevated densities of base substitutions identified 60,309 core SNP sites that represented putative point mutations outside recombination regions. One to 157 putative recombination blocks were identified containing five to 9,514 SNPs. The ratio of SNPs within recombination to the SNPs outside recombination (r/m) was 0,128, indicating a low effect of homologous recombination on the population diversity of *Y. ruckeri*. The core genome SNPs included a remarkable accumulation of non-synonymous variations (*n* = 58,972 SNPs) changing the amino acid sequence and possibly altering protein function. 17,336 SNPs represented silent (synonymous) gene changes, 13,768 SNPs were present in the intergenic regions while 733 SNPs were in other structures such as RNA.

**FIGURE 4:**
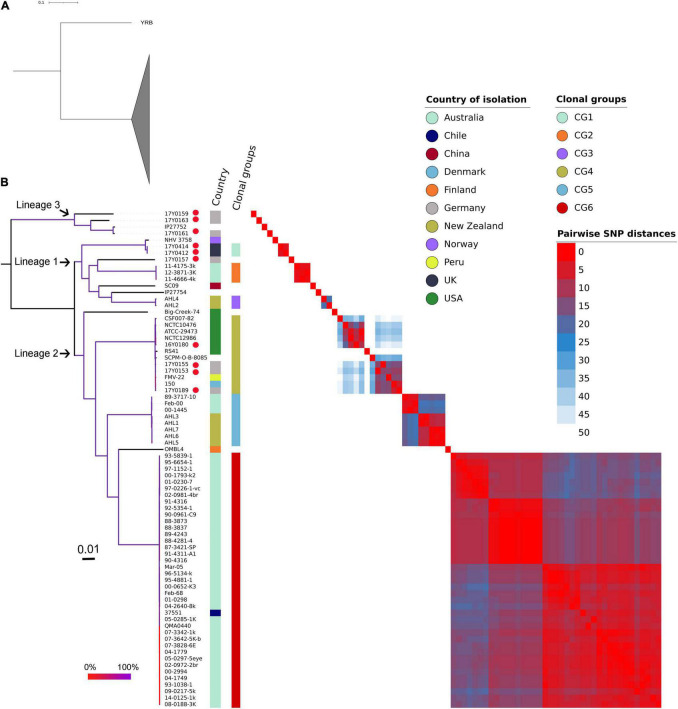
Phylogenetic relationships of the *Yersinia ruckeri* genomes based on whole-genome SNPs after filtering regions with high SNP density using Gubbins. **(A)** Maximum likelihood phylogenetic analysis based on 77 strains showing strain YRB as the most distant to the other investigated *Y. ruckeri* genomes presented with collapsed clade. **(B)** Maximum likelihood phylogenetic tree based on 76 *Y. ruckeri* strains including the 10 strains (red dot) sequenced in this study. The three lineages described in the text are highlighted. Plotted next to the phylogenetic tree are the country of isolation, the clonal groups identified based on less than 50 pairwise SNP, and a heat map showing the SNP distances between each pair of genomes after filtering possibly recombinant regions. The branching pattern was generated by the maximum likelihood method as implemented in the RAxML program. Branch coloration refers to the bootstrap support based on the analysis of 100 resampled trees.

The ML phylogenetic tree generated using RAxML based on non-recombinant SNPs is shown in Figure 4 along with a heat map of pairwise SNP differences between all genomes. The genome of strain YRB was the most distant in the phylogeny. The other strains were grouped into three phylogenetic lineages. Lineage 1 previously described by Barnes et al. (2016) contained strains from Norway, Australia, China, and New Zealand. This lineage included two additional strains from Scotland and one strain from Germany. Three further strains from Germany were placed in lineage 2. Lineage 3 encompassed four strains, three from Germany and one of unknown origin. The German strains exhibited pairwise SNP distances averaging 4,037 SNPs, while the two strains from Scotland displayed only four SNPs.

Grouping the strains on fewer than 50 recombination-purged pairwise SNPs revealed the presence of six clonal groups (CG) with specific geographic associations. CG1 contained strains from Scotland, CG2 from Australia, CG3 from New Zealand, CG5 from both Australia and New Zealand, and CG6 from Australia, with the exception of one strain from Chile. CG4 included strains from the United States, Germany, Peru, and Denmark.

#### Virulence Genes in *Y. ruckeri*

**FIGURE 5:**
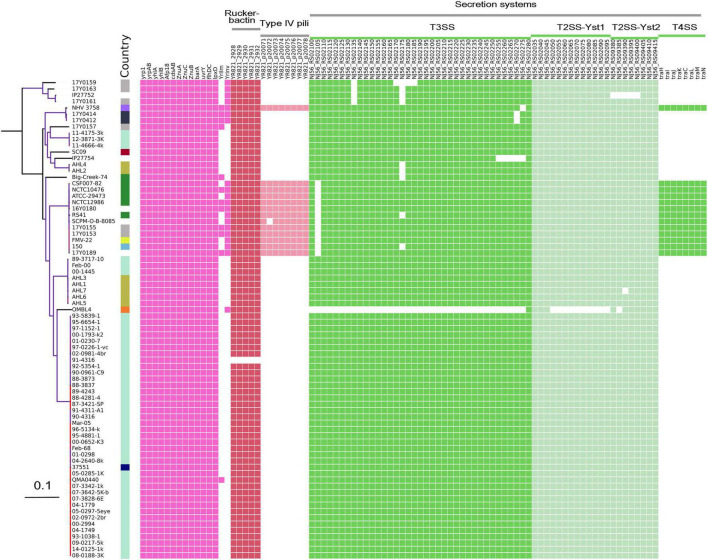
Virulence genes identified in the genomes of *Y. ruckeri*. The presence (colored cells) or absence (white cells) of genes is shown alongside the maximum likelihood phylogeny similar to this figure.

Comparative analysis of virulence genes revealed the presence of a stable set of virulence factors in all *Y. ruckeri* strains (Figure 5). These include toxin genes for the extracellular Yrp1, *Y. ruckeri* peptidases (YrpAB) and *Y. ruckeri* pore forming toxin (yhlBA) as well as the Afp18. Additionally, the *ZnuABC* operon encoding a zinc binding protein and the BarA-uvrY regulator two-component systems were detected in all strains, as well as the cdsAB operon important for cystine transport and utilization, the flhDC operon involved in the regulation of the flagellar secretion system, and *lpxD* involved in lipid A biosynthesis. All these factors have previously been shown to be linked to the pathogenicity of *Y. ruckeri* (Wrobel et al., 2019). The recently described invasion gene *Y. ruckeri* invasin (*yrInv*) (Wrobel et al., 2018a) was detected in 20 strains.

The secretion systems described in strain SC09 included a type III secretion system (T3SS). Two type II secretion systems (T2SS; Yst1 and Yst2) were detected in all strains with the exception of strain OMBL4.

Strains belonging to CG4 as well as strain NHV3758 carried additional virulence factors, the type IV pili system encoded by the *pil* operon and the type IV secretion system encoded by the *tra* operon. As previously reported, these elements are located on a large (80–103 kb) potential virulence plasmid (Wrobel et al., 2018b).

## Discussion

### Exploring the Evolutionary Relatedness of *Y. ruckeri* to the Genus *Yersinia*

The present study performed a comparative analysis on the genomes of the fish pathogen *Y. ruckeri* and explored its phylogenetic relatedness to the further members of the genus *Yersinia*. The results showed that the taxonomic assignment of *Y. ruckeri* could be achieved by different approaches including 16S rRNA gene analysis and whole genome-based methods such as ANI estimation, cgMLST and core genome-phylogenetic analysis. Though these approaches were congruent, analysis of the genus core genome provided a more robust phylogenetic framework for accurate delineation of the species’ genetic relatedness. The large core-nucleotide genome alignment (∼2 Mb) predicted with the Panaroo pipeline provided a matrix for the super resolution phylogenetic analysis of the genus *Yersinia*. Analysis of the classical 16S rRNA marker did not correctly identify the closest relatives to *Y. ruckeri* necessitating careful interpretation of 16S rRNA results. Our results also showed that genome alignments and phylogenetic analyses are more informative and discriminatory than the whole genome allele typing system implemented in the genus-wide cgMLST scheme (Savin et al., 2019). Despite the fact that cgMLST provides portable isolate data easily exchangeable via a centralized database, the application of cgMLST allele typing to define species boundaries can be challenging. The *Yersinia* genus-wide scheme comprises a limited number of loci (500 genes), hence genetic diversity and true inter-strain distances are not well represented above certain thresholds, which may cause problems. The cgMLST system is very sensitive to assembly artifacts by falsely increasing allelic distances. Our results show that the phylogenetic analysis of core genome alignments is a robust approach that complements the average nucleotide identity results (Chung et al., 2018). The combination of both methods provides a stable scheme for delineating *Yersinia* species. The sole application of allele typing or 16S rRNA gene marker analysis requires caution during data interpretation.

Genomic analyses of bacterial pathogens revealed variable evolutionary processes that possibly influenced bacterial lifestyle and led to specialization in specific ecological niches. *Y. ruckeri* has arguably been discussed as a fish-restricted pathogen but the underlying genetic mechanisms and signatures of adaptation remain to be elucidated. In many bacteria, expansions of repetitive insertion sequences, large-scale chromosomal rearrangements and the high frequency of pseudogenes have been associated with narrow niche specialization as seen in e.g., *Shigella* (Feng et al., 2011), *Streptococcus agalactiae* (Almeida et al., 2016), and *Y. pestis* (Reuter et al., 2014). Although genomic analyses on the structure and layout of genes in *Y. ruckeri* revealed conservation and low incidence of chromosomal rearrangements, *Y. ruckeri* showed various mobile genetic elements. Moreover, *Y. ruckeri* displayed pseudogenization in the form of gene fragmentation and truncation most likely due to internal stop codons. These genetic changes possibly disrupt gene transcription and translation, contributing to genome streamlining via removal of genes whose functions are no longer required (Feng et al., 2011). However, in *Y. ruckeri*, pseudogenes and insertion sequences were not as pronounced as in other *Yersinia* spp. notably *Y. pestis* (Chen et al., 2010), indicative of a different evolutionary pathway. The small genome size of *Y. ruckeri* relative to other *Yersinia* species is similar to other aquatic bacteria with reduced functional repertoire. Previous studies corroborate our findings, e.g., the absence of genes encoding for urease, methionine salvage, B12-related metabolism and myo-inositol degradation in *Y. ruckeri* (Chen et al., 2010; Tan et al., 2016).

The pattern of gene presence/absence in the accessory genome was in close agreement with the core genome phylogeny, possibly indicating concurrent co-evolution of *Yersinia* core genomes with acquisition and loss of genes. This is in agreement with a previous study (Tan et al., 2016) describing limited influence of lateral gene transfer on *Yersinia* phylogeny. In our study, we performed a GWAS analysis to identify differentially present genes in 100 representative *Yersinia* genomes. Although the selected genomes represented all validated *Yersinia* species, they may not have captured the diversity of this genus. Nevertheless, our analyses revealed the distinctive presence of 303 additional genes in *Y. ruckeri*. These genes were predicted to encode (1) virulence factors such as Afp18, Yrp1 and phage proteins and (2) metabolic enzymes involved in the utilization of iron, amino acids, specifically arginine and histidine, as well as carbohydrates. These factors are involved in the pathogenesis of ERM. Most of these genes were arranged in cassettes, which may prevent random dispersal throughout the genome. The association of these cassettes with mobile elements are indicative of horizontal acquisition. These results could indicate that a process of gene acquisition may have contributed to the evolution of the species.

### Genome Comparison of *Y. ruckeri* Strains

The genome of *Y. ruckeri* is highly conserved in terms of gene structure, gene layout and functional categorization of genes. It harbors a large core genome, representing 85% of the total number of genes. Similar to other *Yersinia* species (Tan et al., 2016), diversification of the species core genome was mainly due to gene mutations. The pangenome is open with an estimated contribution of 11 new genes at the 77th sequenced genome (Supplementary Figure 4B). Our results corroborate and consolidate the findings of Barnes et al. (2016) by adding further strains to the analysis (total = 77) and the calculation of proposed regression models (Tettelin et al., 2008). It is very likely that the pangenome variation has occurred due to the variable mobile elements and plasmids in the strains. *Y. ruckeri* is thought to be vulnerable to the acquisition of genetic elements due to the absence of CRISPR systems (Barnes et al., 2016).

Recent studies based on whole genome sequence analysis and multilocus variable-number tandem-repeat analysis (Barnes et al., 2016; Gulla et al., 2018) described geographical endemism for numerous genetically distant lineages of *Y. ruckeri* strains. To date a large-scale analysis on *Y. ruckeri* strains from Germany is missing. However, our results allocate the German strains to three genetically distant phylogenetic lineages of the species. A possible explanation for the circulation of different lineages in Germany might be the acquisition of breeding stock and fish spawn from different geographic origins; it may also explain the presence of German strains (*n* = 3) in the clonal group (CG4) together with strains from the United States, Peru, and Denmark. Gulla et al. (2018) made similar observations using MLVA, pointing to a possible spread of *Y. ruckeri* strains from North America to Europe and South America.

The chromosomal background of this CG4 might have conferred a selective advantage toward acquisition and maintenance of virulence plasmids. CG4 contains a 103.8 kb large plasmid similar to the pYR3 plasmid previously described (Nelson et al., 2015). This plasmid and the 80.8 kb plasmid (pYR4) of strain NVH_3758 belong to the IncFII plasmid family (Méndez et al., 2009; Wrobel et al., 2018b); it contains several mobile protein elements and a conjugative system. A large region of ∼55 kb is present in both plasmids including a *pil* operon encoding for a type IV pilus and several *tra* operons encoding for the T4SS. The *tra* operon is important for the virulence of *Y. ruckeri* as previously demonstrated (Méndez et al., 2009). The association between the type of virulence plasmid and phylogenetic group can be explained by the clonal expansion of hypervirulent strains as a result of horizontal plasmid acquisition. On the other hand, our results show that similar virulence genes are present in the majority of the analyzed *Y. ruckeri* strains. Previous studies indicated at differences in ERM pathogenicity between *Y. ruckeri* strains (Guijarro et al., 2018). This could imply a multifactorial nature of ERM, in which environmental factors may be involved and influence the transcription and regulation of virulence genes. It can also be hypothesized that strain fitness in terms of its metabolic repertoire may have an important role in the pathogenicity of ERM in fish.

## Conclusion

This study provides valuable insights into the genomic variability and phylogenetic relatedness of *Y. ruckeri*. Our results show that the genetic relatedness of the species is better delineated using core genome alignment followed by phylogenetic analysis rather than genome-wide allele typing or 16S rRNA gene analysis. Phylogenetically, *Y. ruckeri*, *Y. entomophaga*, and *Y. nurmii* form a species complex that is distinct from other *Yersinia* species. *Y. ruckeri* has unique genomic regions that might play a role in the pathogenesis of ERM. *Y. ruckeri* show highly homogeneous genomes with a high degree of synteny and contain numerous genomic islands but lack the CRISPR-Cas system. Limited genomic and virulence plasticity was observed arising from variable components of mobile elements and plasmids. Strain grouping based on the ML phylogeny revealed the absence of geographic clustering of several *Y. ruckeri* strains indicating a possible dissemination of a specific strain group. The German *Y. ruckeri* strains belong to the three genetically distant phylogenetic lineages.

## Materials and Methods

### Cultivation and Identification of *Y. ruckeri* Strains

Strains were routinely cultured on Columbia blood agar at 25°C. Initial identification was done using matrix-assisted laser desorption ionization-time of flight mass spectrometry (MALDI-TOF MS; Bruker Daltonik GmbH, Bremen, Germany) and API 20E (Biomerieux, Nürtingen, Germany). Typing was done by Pulsed Field Gel Electrophoresis (PFGE) with *Not*-I, Dice similarity coefficient and UPGMA. In addition, repetitive sequence-based PCR assays were performed, including BOX-AIR-based repetitive extragenic palindromic-PCR (BOX-PCR), (GTG)5-PCR, enterobacterial repetitive intergenic consensus (ERIC-PCR), and repetitive extragenic palindromic PCR (REP-PCR) (Huang et al., 2013).

### *Y. ruckeri* Genome Sequencing

Genomic DNA was extracted using the QIAGEN Genomic-tip 100/G kit and the Genomic DNA Buffer Set (QIAGEN GmbH, Hilden, Germany) following the manufacturer’s instructions. DNA quality was assessed with a Qubit 3 Fluorometer using the Qubit^TM^ dsDNA HS Assay Kit (Invitrogen^TM^, Germany). Whole genome sequencing was performed on the Pacific Biosciences RS sequencer with SMRT Technology PacBio RS II with SMRT Technology PacBio RS II (Pacific Biosciences, Menlo Park, CA, United States) at GATC (Konstanz, Germany), using standard protocols according to the manufacturer’s instructions. Paired-end sequencing was performed using an Illumina MiSeq instrument. Sequencing libraries were created using the Nextera XT DNA Library Preparation Kit (Illumina Inc., United States) to generate reads of 300 bp in length according to the manufacturer’s instructions.

### Genome Assembly, Circularization and Polishing

*De novo* genome assembly and circularization for long sequencing reads was performed as previously described (Abdel-Glil et al., 2021). Briefly, RS_HGAP_Assembly v3 available via SMRT Analysis system v2.3.014 was used for the assembly of the PacBio data (Chin et al., 2013). Gepard v1.40 was then used to identify similar parts at the ends of each contig (Krumsiek et al., 2007). Genome circularization was performed by merging overlapping ends of each genome using Circlator v1.5.0 or check_circularity.pl from SPRAI (Hunt et al., 2015). Quiver algorithm (RS.Resequencing.1) was iteratively used for error corrections in the merged region (Chin et al., 2013). For additional polishing of the genome assemblies, Pilon v1.23 was applied four times using the Illumina data to correct the final assembled data with standard settings (Walker et al., 2014).

### Genome Annotation and Functional Categorization of Genes

Assembled genomes were annotated using the rapid genome annotation pipeline Prokka v1.13.3 (Seemann, 2014). For prediction of CRISPR sequences, the CRISPR Recognition Tool v1.1 (Bland et al., 2007) was used in default mode. Prophage sequences were predicted by querying contigs assembled to the prophage databases implemented in the PHASTER web service (Arndt et al., 2016). Predicted prophages were classified as intact, questionable or incomplete using the scoring system described by Arndt et al. (2016). For prediction of insertion elements, ISEscan v1.7.2 software (Xie and Tang, 2017) was used with the “–removeShortIS” flag enabled to report only complete IS elements, i.e., IS elements with terminal inverted repeat sequences and longer than 400 bp. The circularized *Y. ruckeri* plasmids were separated and analyzed for their degree of homology by applying pyani v0.2.3 software (Pritchard et al., 2016) to report the average nucleotide identity values, percentage of genome aligned and the Hadamard distance matrix. The latter merges the average nucleotide identity values and the percentage of the aligned genomic regions. Pyani implements alignment algorithms for ANI calculation. Here, the BLAST algorithm was used for alignment.

Reference based identification of potential pseudogenes was done with Pseudofinder v1.0^1^. The non-redundant NCBI protein database was used as reference database for Pseudofinder, and diamond 0.9.24 (Buchfink et al., 2015) for high throughput protein alignment. Functional annotation of genes using orthology assignment was performed using eggNOG-mapper v2.0 (Huerta-Cepas et al., 2018). Chromosome alignment of orthologous and xenologous regions of the *Y. ruckeri* strains was performed using progressive Mauve algorithm applied in standard mode (Darling et al., 2010).

### Genome Comparison and Phylogenetic Analysis of the *Yersinia* Genus

For genus-wide comparison, barrnap v0.9^2^ was used to predict and extract the rRNA genes. Extracted 16S rRNA sequences were aligned using MAFFT v7.307 (Katoh and Standley, 2013) followed by an ML phylogenetic analysis using RAxML v8.2.10 (Stamatakis, 2014) with the general time-reversible (GTR)-gamma model and 100 bootstrap replicates.

Estimation of the gANI between the 100 *Yersinia* genomes was done using pyani v0.2.3 (Pritchard et al., 2016), using standard parameters. ANI values were estimated by applying two independent methods: FastANI v1.3 which uses the kmer content for alignment-free sequence mapping (Jain et al., 2018), and the MiSI (Varghese et al., 2015) tool “ANIcalculator v1,” which excludes RNA genes and reports alignment fractions and ANI values based on the orthologous protein-coding genes identified as bidirectional best hits.

To identify the genus core genome, we used Panaroo v1.2.7 to identify the core orthologous genes of the 100 *Yersinia* genomes using the stringency mode “strict” (Tonkin-Hill et al., 2020). The default thresholds of Panaroo were applied including sequence identity at 98% and core genome assignment at 95%. Gene-by-gene alignment was done with MAFFT v7.307 as in Panaroo. Concatenated core gene alignment was then used for ML phylogenetic analysis using RAxML v8.2.10 as mentioned above.

The cgMLST allele typing for the 100 *Yersinia* strains was predicted by submitting the genome sequences to the *Yersinia* cgMLST scheme using the Pasteur MLST database^3^ (Savin et al., 2019). Next, the Genome Comparator plugin of BIGSdb implemented in the Pasteur database was used for genome comparison. The resulting allelic profiles were visualized in a minimum spanning tree using the online version of Grapetree software (Zhou et al., 2018).

Pan-GWAS analysis was done with Scoary v1.6.10 (Brynildsrud et al., 2016) to identify the statistical association between the genus accessory genes and the *Y. ruckeri* species. We used the accessory genes defined with the Panaroo program as input for Scoary. A total of 10,000 permutation replicates was applied using genes with 100% sensitivity and specificity, and with *P*-values of less than 10^–5^ (naïve, Benjamini–Hochberg-adjusted and Bonferroni). To confirm the specificity of the identified genes for *Y. ruckeri*, we applied BLAST analysis to the nucleotide sequences at 90% sequence identity and 80% coverage. Functional annotation of the genes was done using the COG database as described above. Furthermore, PSORTb version 3.0.3 was used for subcellular localization prediction (Yu et al., 2010).

### Genome Comparison and Phylogenetic Analysis for *Y. ruckeri* Strains

For species-wide comparison, 77 *Y. ruckeri* genomes were included. Pangenome analysis was performed as mentioned above using the Panaroo pipeline (Tonkin-Hill et al., 2020). PanGP v1.0.1 program (Zhao et al., 2014) was used for the analysis of pangenome profiles with a distance guide algorithm of 100 replicates and 1000 permutations of genome order.

Parsnp v1.2 (Treangen et al., 2014) performed a core genome alignment with default settings. Filtering regions with high SNP density from the alignment was done with Gubbins v2.4.1 in standard mode (Croucher et al., 2015). RAxML v8.2.10 was applied as mentioned above to construct ML phylogenetic trees (Stamatakis, 2014). Phylogenetic trees were visualized using iTOLv6 (Letunic and Bork, 2019).

A search for the virulence related genes in *Y. ruckeri* was performed using BLASTN v2.10.1+ (Altschul et al., 1997) as featured in ABRicate v1.0.1^4^. A sequence identity threshold of 80% and coverage threshold of 80% was applied.

## Data Availability Statement

The original contributions presented in the study are publicly available. These data can be found in the NCBI under BioProject PRJNA767598 (https://www.ncbi.nlm.nih.gov/search/all/?term=PRJNA767598).

## Author Contributions

MA-G: bioinformatic data analyses and manuscript draft. UF, UM, DS, and LS: cultivation and microbial analysis of *Y. ruckeri* and manuscript revision. HN and LS: project planning, data analysis, and manuscript revision. All authors contributed to the article and approved the submitted version.

## Conflict of Interest

The authors declare that the research was conducted in the absence of any commercial or financial relationships that could be construed as a potential conflict of interest.

## Publisher’s Note

All claims expressed in this article are solely those of the authors and do not necessarily represent those of their affiliated organizations, or those of the publisher, the editors and the reviewers. Any product that may be evaluated in this article, or claim that may be made by its manufacturer, is not guaranteed or endorsed by the publisher.

## Footnotes

1
https://github.com/filip-husnik/pseudofinder

2
https://github.com/tseemann/barrnap

3
https://bigsdb.pasteur.fr/yersinia/

4
https://github.com/tseemann/abricate

## Supplementary Material

Supplementary Figure 1 Representation of the circular chromosome of the *Yersinia ruckeri* type strain (3,696,254 bp). Circles inward represent predicted annotated coding genes in clockwise (1) and anticlockwise (2) orientations with genes colored according to the predicted clusters of orthologous classes as shown in the legend. Circle 3 in black represents the GC content. Circle 4 shows histogram plots for positive (purple) and negative (green) GC skews. Positive skew indicates a higher presence of G than C while negative skew indicates more C than G based on the equation (G−C)/(G+C) calculated over a window of 10 kb.

Supplementary Figure 2 Phylogenetic relationship within the *Yersinia* genus based on 16S rRNA sequences. The branching pattern was generated by the maximum likelihood method as implemented in the RAxML program. The tree is midpoint rooted. Branch coloration refers to the bootstrap support from zero (red) to 100 (violet) based on the analysis of 100 resampled trees. Collapsed nodes include taxa of a single species.

Supplementary Figure 3 Results of the average nucleotide identity (ANI) analysis. **(A)** A heat map showing the ANI values calculated over the base pair matches of the aligned regions between each pair of genomes using pyani (Pritchard et al., 2016); **(B)** Histogram of the ANI values between each genome pair produced with bactaxR (Carroll et al., 2020); **(C)** Heat map showing the alignment coverage for the calculation of nucleotide identity; the dendrograms shown above and on the left side of the heat maps are based on hierarchical clustering of ANI values using the simple linkage method as described (Pritchard et al., 2016).

Supplementary Figure 4 Pangenome analysis encompassing 77 *Yersinia ruckeri* genomes. **(A)** Plot showing the trajectory pattern of expansion of pangenome genes (blue curve) versus the reduction of the core genome (green curve). The plot depicts the total number of pan and core genes per each sequenced genome. **(B)** Plot representing the contribution of new genes to the overall gene pool with sequential addition of genomes.
