# Supplementary figures and images for: Phylogenetic Relatedness and Genome Structure of Yersinia ruckeri Revealed by Whole Genome Sequencing and a Comparative Analysis
Source: Front Microbiol. 2021 Nov 18;12:782415. doi: 10.3389/fmicb.2021.782415 (PMC8640586; doi:10.3389/fmicb.2021.782415)

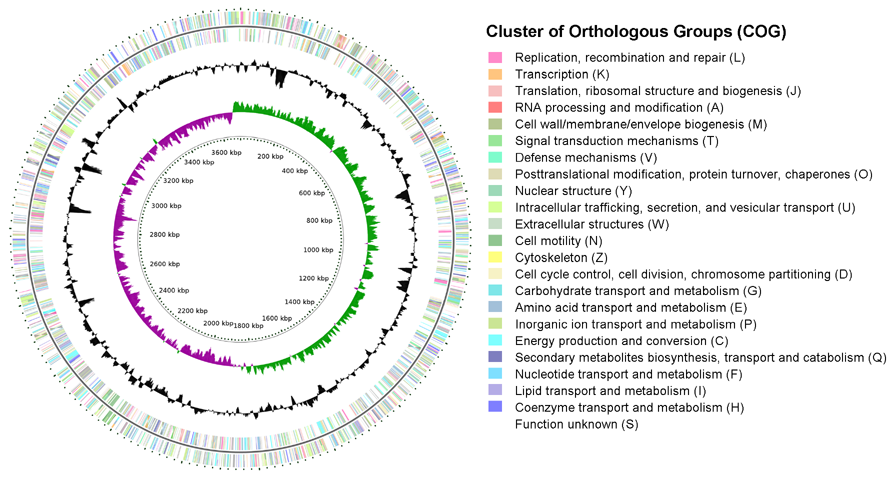

Supplement: Supplementary Figure 1 — Representation of the circular chromosome of the Yersinia ruckeri type strain (3,696,254 bp). Circles inward represent predicted annotated coding genes in clockwise (1) and anticlockwise (2) orientations with genes colored according to the predicted clusters of orthologous classes as shown in the legend. Circle 3 in black represents the GC content. Circle 4 shows histogram plots for positive (purple) and negative (green) GC skews. Positive skew indicates a higher presence of G than C while negative skew indicates more C than G based on the equation (G−C)/(G+C) calculated over a window of 10 kb. [file Image_1.TIF]

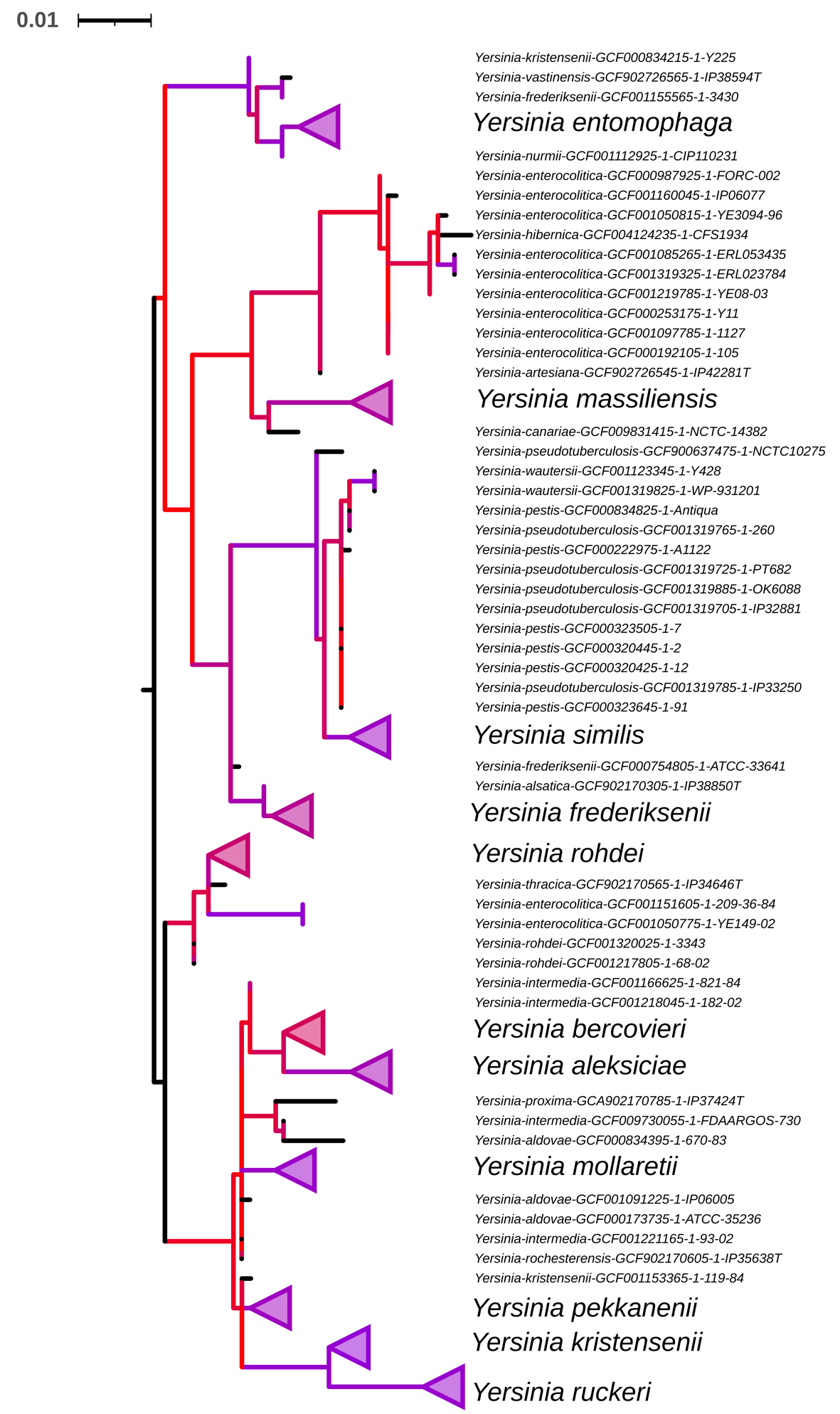

Supplement: Supplementary Figure 2 — Phylogenetic relationship within the Yersinia genus based on 16S rRNA sequences. The branching pattern was generated by the maximum likelihood method as implemented in the RAxML program. The tree is midpoint rooted. Branch coloration refers to the bootstrap support from zero (red) to 100 (violet) based on the analysis of 100 resampled trees. Collapsed nodes include taxa of a single species. [file Image_2.TIF]

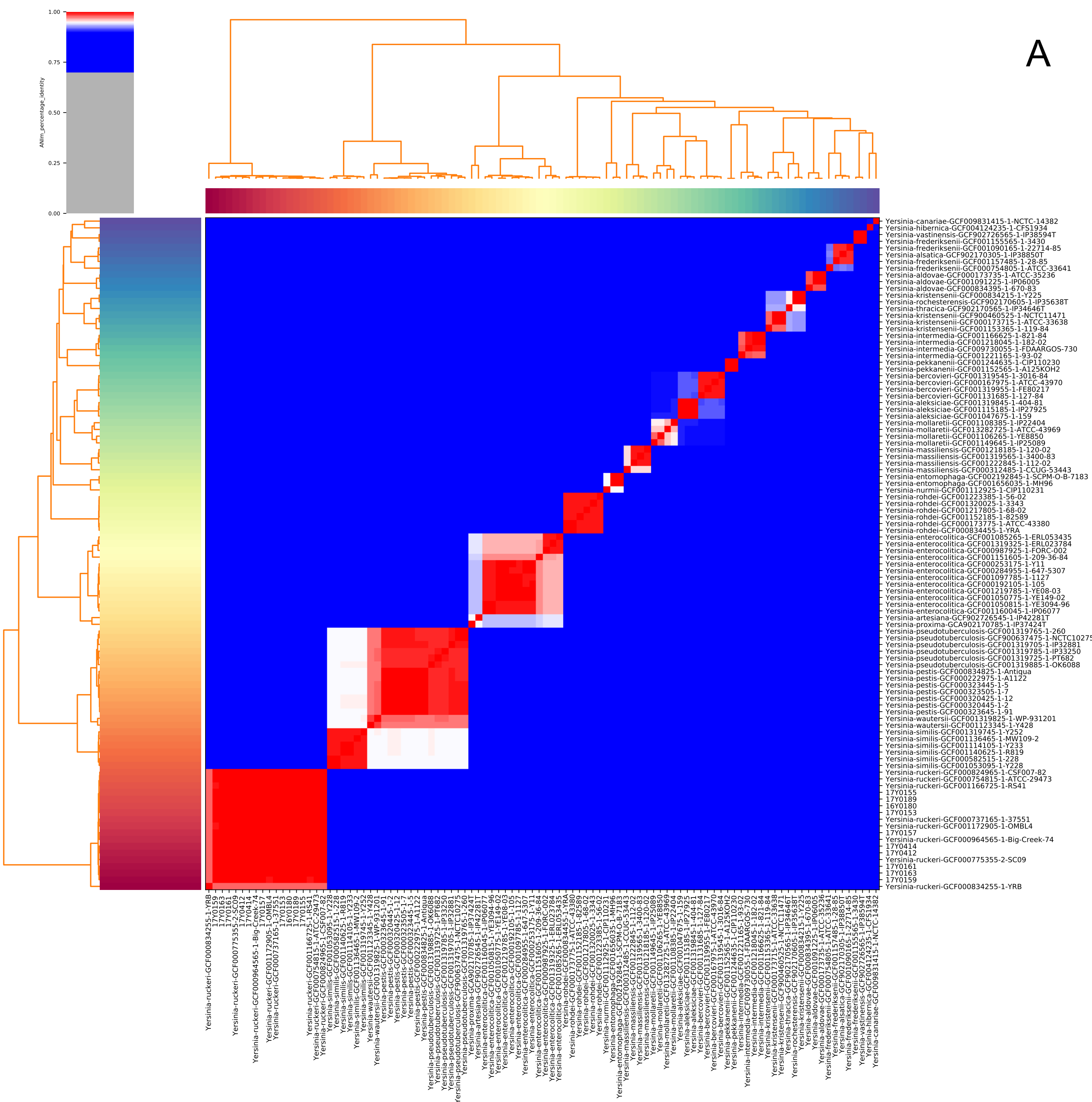

A

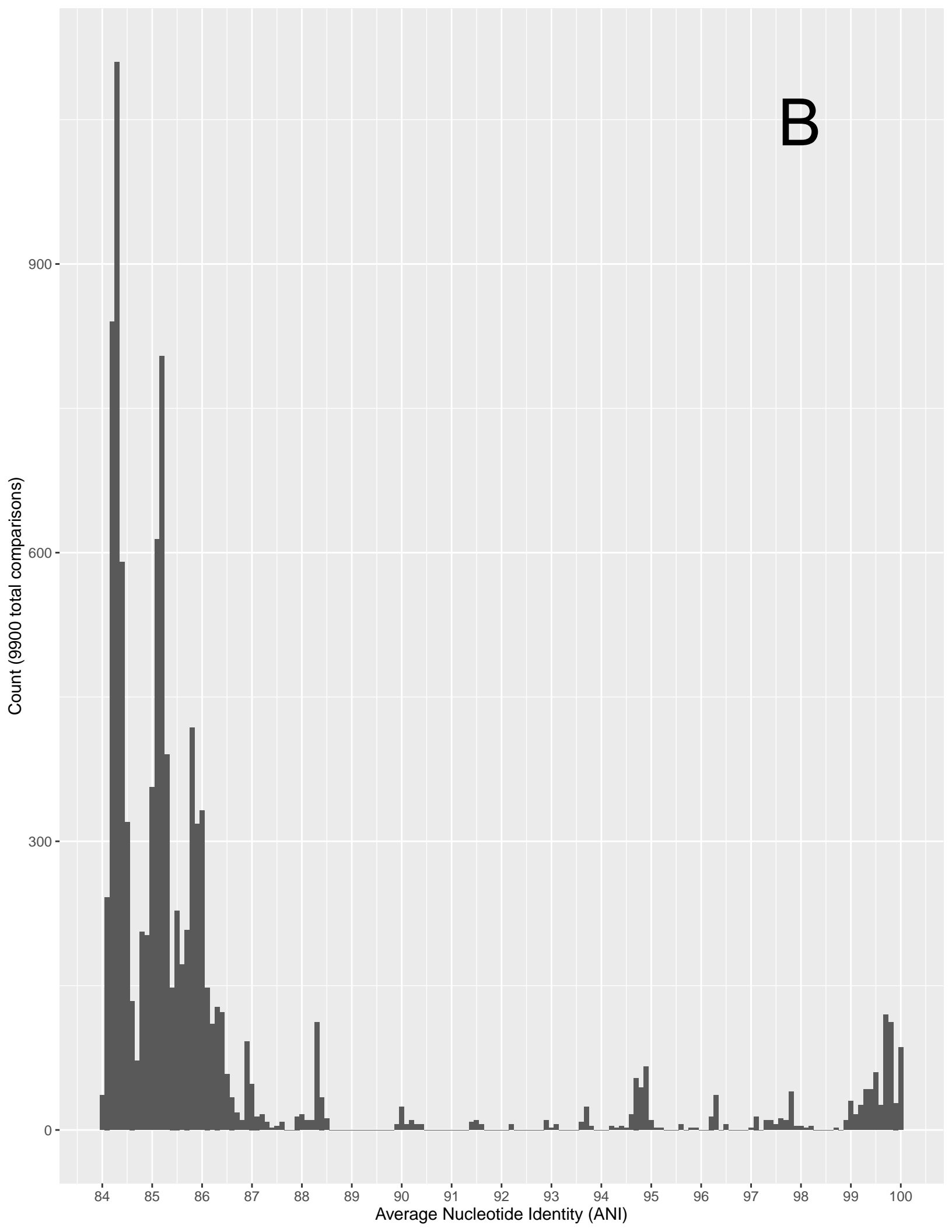

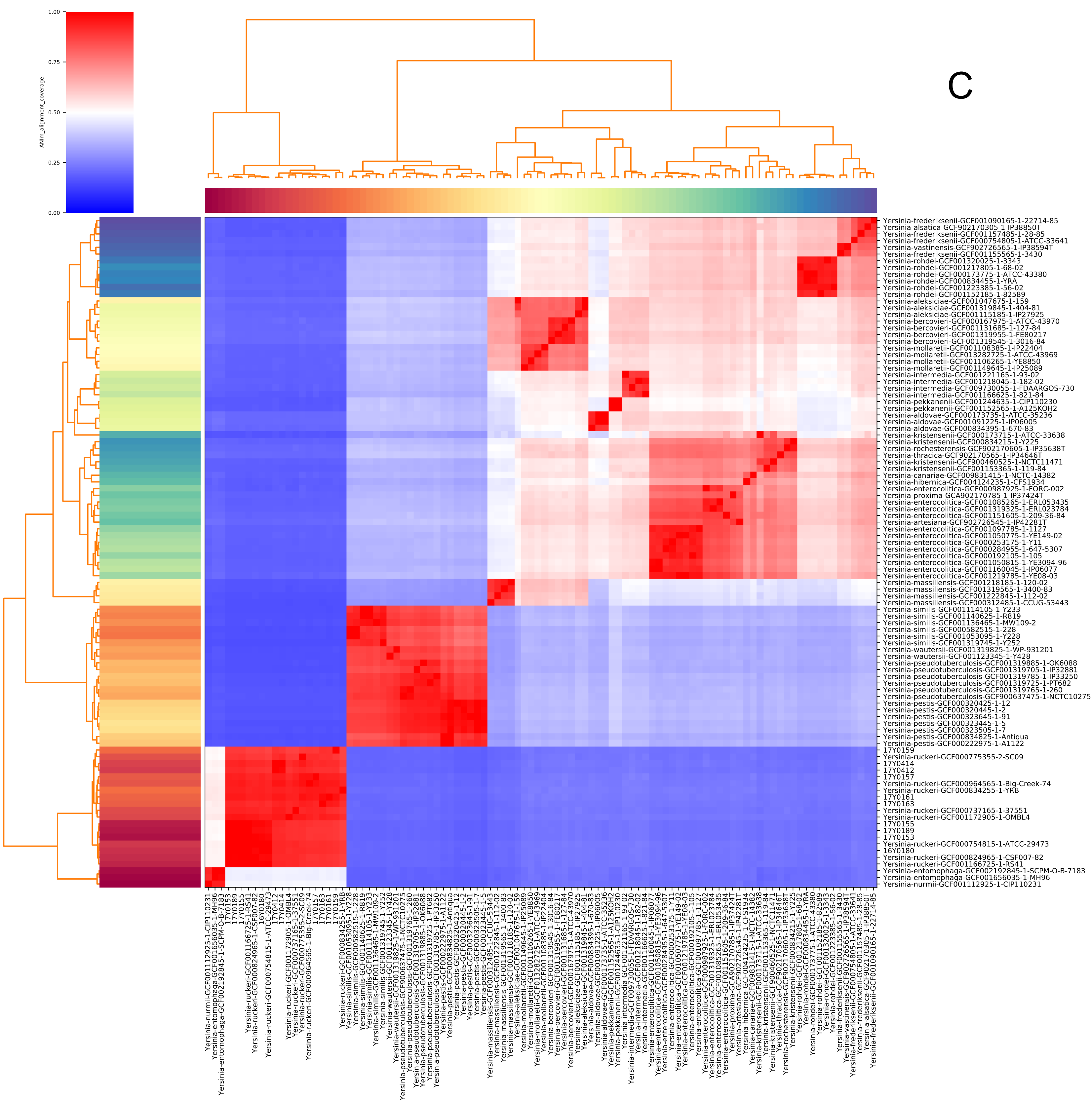

Supplement: Supplementary Figure 3 — Results of the average nucleotide identity (ANI) analysis. (A) A heat map showing the ANI values calculated over the base pair matches of the aligned regions between each pair of genomes using pyani (Pritchard et al., 2016); (B) Histogram of the ANI values between each genome pair produced with bactaxR (Carroll et al., 2020); (C) Heat map showing the alignment coverage for the calculation of nucleotide identity; the dendrograms shown above and on the left side of the heat maps are based on hierarchical clustering of ANI values using the simple linkage method as described (Pritchard et al., 2016). [file Image_3.PDF]

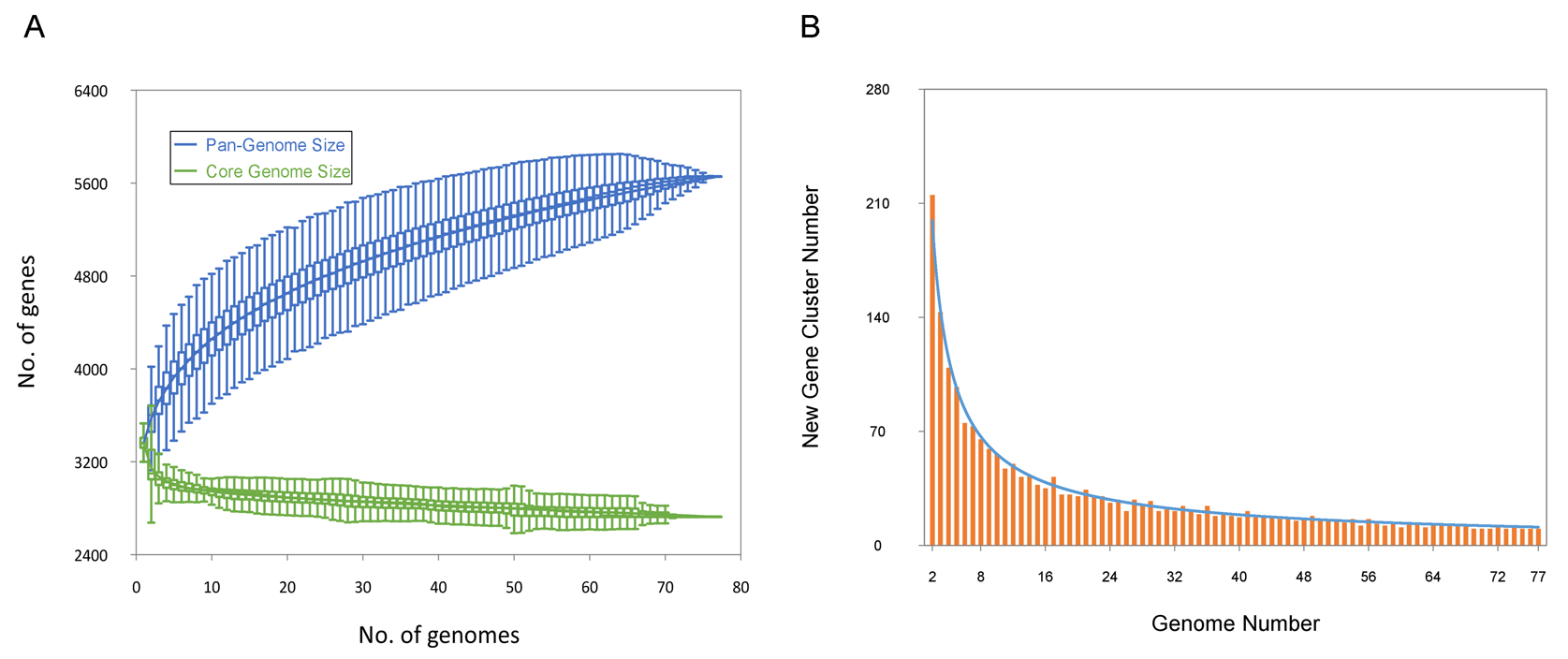

Supplement: Supplementary Figure 4 — Pangenome analysis encompassing 77 Yersinia ruckeri genomes. (A) Plot showing the trajectory pattern of expansion of pangenome genes (blue curve) versus the reduction of the core genome (green curve). The plot depicts the total number of pan and core genes per each sequenced genome. (B) Plot representing the contribution of new genes to the overall gene pool with sequential addition of genomes. [file Image_4.TIF]
